# Estimating the standardized incidence ratio (SIR) with incomplete follow-up data

**DOI:** 10.1186/s12874-017-0335-3

**Published:** 2017-04-12

**Authors:** Heiko Becher, Volker Winkler

**Affiliations:** 1 University Medical Center Hamburg-Eppendorf, Martinistr. 52, 20246 Hamburg, Germany; 2 Institute of Public Health, University of Heidelberg, Im Neuenheimer Feld 324, 69120 Heidelberg, Germany

**Keywords:** Epidemiological methods, Cohort studies, Incidence, Missing data, Standardized incidence ratio

## Abstract

**Background:**

A standard parameter to compare the disease incidence of a cohort relative to the population is the standardized incidence ratio (SIR). For statistical inference is commonly assumed that the denominator, the expected number of cases, is fixed. If a disease registry is available, incident cases can sometimes be identified by linkage with the registry, however, registries may not contain information on migration or death from other causes. A complete follow-up with a population registry may not be possible. In that case, end-of-follow-up date and therefore, exact person-years of observation are unknown.

**Methods:**

We have developed a method to estimate the observation times and to derive the expected number of cases using population data on mortality and migration rates. We investigate the impact of the underlying assumptions with a sensitivity analysis.

**Results:**

The method provides a useful estimate of the SIR. We illustrate the method with a numerical example, a simulation study and with a study on standardized cancer incidence ratios in a cohort of migrants relative to the German population. We show that the additional variance induced by the estimation method is small, so that standard methods for inference can be applied.

**Conclusions:**

Estimation of the observation time is possible for cohort studies with incomplete follow-up.

**Electronic supplementary material:**

The online version of this article (doi:10.1186/s12874-017-0335-3) contains supplementary material, which is available to authorized users.

## Background

The analysis of epidemiologic cohort studies usually requires person-years (py) calculation to measure the exact time at risk. Person-years are not only used to compute different rates within a cohort resulting in e.g. rate ratios (RR), but also to calculate the commonly applied standardized incidence or mortality ratio (SIR, SMR) to compare the observed number of certain events with the expected number given the rates in the underlying population [1]. A SIR or a SMR estimates the occurrence of an event in a population relative to what might be expected if the population had the same experience as some larger comparison population designated as ‘normal’ or average or reference.

To be able to calculate the exact accumulated number of person-years within a cohort, the dates (i) begin of follow-up and (ii) end of follow-up must be known for each individual. For estimating the SIR, the date of disease onset and reference incidence rates for comparison (to calculate the expected number of cases) must also be available. The latter may come from disease registries, e.g. a cancer registry. The end of the observation period is defined as the minimum of date of death, date of disease onset and end-of-follow-up/lost-to-follow-up date.

Statistical inference for the SIR and the SMR is usually based on the assumption that the denominator is fixed and that the numerator is Poisson distributed. Both approximate and exact solutions have been developed [1]. The analysis is implemented in common software programs like SAS, R or STATA. Few authors, however, have considered the case when the denominator must be considered as random. Silcocks showed that the beta distribution can be used for statistical inference when the expected number of events is subject to random variation [2]. More recently, Beyene and Moineddin compare methods for measuring the “location quotient”, which is the relative contribution of a rate in a subset (size n_i_) to the whole population (size n) [3]. In both of these cases the variance of the denominator cannot be neglected because the underlying population is large enough relative to the study population.

In the present case, a different situation is considered which has not been paid much attention. While the linkage of a cohort with a disease registry may be straight forward, the linkage with the population registry may be hampered by bureaucracy, data protection issues, costs, or other reason. In that case the exact person-years are unknown. This was the case for a cohort study conducted by us and motivated the present work.

In this paper we present a method to estimate the SIR and to perform statistical inference under the following situation: (i) The cohort is fully defined and information on basic covariates (date of birth, date of entry into cohort, sex, covariates where appropriate) is complete (ii) incident cases of the disease(s) of interest have been identified through linkage with a disease registry, with date of disease onset known. This registry may cover the geographical area from which the cohort originated, but not necessarily the whole country (iii) a mortality follow-up is not available, i.e. it is unknown if a person died of a disease other than the disease of interest, and it is unknown whether and when a person moved out of the catchment area of the disease registry.

This situation is not uncommon. In the second section, we provide the estimate, a procedure to implement this in SAS, an estimate for the variance, and give recommendations for a sensitivity analysis. In the third section, we illustrate the procedure with a simple artificial example.

In the fourth section we investigate properties of the estimation procedure with a simulation study. In the fifth section, we present data from a cohort study in which this situation occurred [4]. It is a study on immigrants from the Former Soviet Union in a German state (Saarland) where we calculated the SIR for cancer, based on linkage with the Saarland cancer registry. This study was performed with 18 621 migrants from the Former Soviet Union who immigrated between 1990 and 2005 to the German federal state of Saarland. For every individual, name, sex, date of birth, date of immigration (start of the observation period), and first city of residence was available. A complete incidence follow-up was done by linking the cohort with the Saarland cancer registry which is a high quality register for this federal state [5]. 470 incident cancer (except non-melanoma skin cancer) cases were identified. Mortality follow-up and tracing of individuals who relocated from the state, however, was not performed. We present a sensitivity analysis to assess the effect of the assumptions made in the method. Finally, we discuss the relevance of our findings.

## Methods

### Outline and notations

**Fig. 1:**
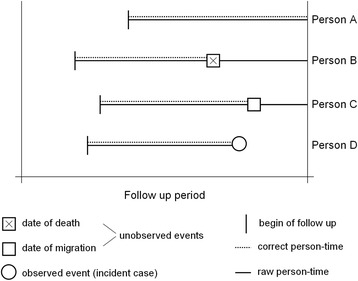
Illustration of possible events during a follow-up procedure for individuals entering the cohort at variable dates

We consider data of a cohort with N individuals numbered as *n =* 1, …, N where date of birth, date of beginning of follow-up, sex and possibly other covariates are known. We assume further that a disease registry exists which covers the geographical area from which the cohort was selected. Linkage with this disease registry yields the date of diagnosis for those diseased before a fixed follow-up date. Then, for each individual the date of diagnosis for those diagnosed within the catchment area of the registry (in the following: study area) or no information on the end of follow-up is given. Thus, four events are possible for each individual as given in Fig. 1.

Event 1) person was identified as incident case (person D)

Event 2) person died from a cause other than the disease of interest at date d_i_ (person B)

Event 3) person moved out of the study area at date d_i_ (person C)

Event 4) person is alive at the end of follow-up date and residing in the study area (person A)

Events of person B and C are not observed. An assumption that all these individuals contribute person-years until the end-of-follow-up date is incorrect and would overestimate the person-years and hence underestimate the SIR. The SIR for a disease D is estimated by the ratio of observed O and expected cases E. E is calculated as

$$ E=\kern0.5em {\displaystyle \sum_{i, j} p{y}_{i\kern0.1em  j}\kern0.5em {\lambda}_{i\kern0.1em  j}} $$

where *py*_*ij*_ are the observed person-years in the full cohort for age group *i* and calendar-period *j*, and λ_ij_ the corresponding incidence rates for disease D in the reference population.

For the estimation according to the later proposed method we need the mortality rates for all other diseases except D, and the out-migration rates. Both may be age- and period-dependent, and we denote these as *μ*_*ij*_ and *ν*_*ij*_ , respectively.

To introduce the method for person-years estimation, we also need the estimated person-year contribution of an individual *n* for year 1, 2 … . We denote this as ${\hat{py}}_{i1 n}$, ${\hat{py}}_{i2 n}$ etc. Year 1 denotes the first calendar year in which cohort members contribute observation time.

### Person-years estimation with incomplete follow-up for a single individual

In order to estimate py_ij_ from the available data, we assume that mortality rates for causes other than the disease of interest μ_ij_ which can be applied to the cohort (by age and calendar period) (event 2) and the migration rates ν_ij_ (event 3) are available. Note that the following applies to the cohort members who did not become incident cases during the follow-up. For these incident cases, person-times are exactly known and can be assigned to age groups and calendar periods as usual.

For sake of simpler presentation we assume for a moment that follow-up start and birthday is January 1^st^. We assume further for a moment that age groups and calendar periods of one year are considered, that the expected person-year contribution for an individual *n* for which one of the events occurred in a year is 0.5 years, and that the events 1,2,3 are independent. Then, the estimated person-year contribution for individual n which is not an incident case belonging to age *i* at calendar year 1 after entry into the study, ${\hat{py}}_{i1 n}$, is

$$ {\hat{py}}_{i1 n}=\left(1-{\mu}_{i1}\right)\left(1-{\nu}_{i1}\right)+0.5\left({\mu}_{i1}+{\nu}_{i1}-{\mu}_{i1}{\nu}_{i1}\right)=\left(1-{\gamma}_{i1}\right)+0.5{\gamma}_{i1} $$

With

1 $$ {\gamma}_{i1}={\mu}_{i1}+{\nu}_{i1}-{\mu}_{i1}{\nu}_{i1} $$

We can assume small rates, so *γ*_*i*1_ can be approximated by *γ*_*i*1_ = *μ*_*i*1_ + *ν*_*i*1_. So if, for example, *μ*_*i*1_ = 0.002 and *ν*_*i*1_= 0.013, then *γ*_*i*1_ = 0.002 + 0.013 − 0.002 × 0.013 = 0.015 − 0.000026 = 0.014974 ≈ 0.015, and an estimate for the person-year contribution in the first year is (1–0.015) + 0.5 × 0.015 = 0.9925 person-years instead of a full person-year. In other words, from 1000 individuals in that age group who did not get the disease of interest, an expected number of 15 individuals either die from another disease or migrate within a year. Each of these 15 individuals contribute an expected half person-year (since a uniform distribution of the time of occurrence of the events within a year can be assumed), so the expected total number of person-years for these 1000 individuals is 985 + 15x0.5 = 992.5 person-years, i.e. 0.9925 per person. For year 2 calculation is similar: From 1000 individuals in that age group who did not get the disease of interest, an expected number of 15 either died from another disease or migrated in the first year and therefore do not contribute person-time for the second year. From the remaining 985 individuals, 985 × 0.015 = 14.775 are expected to die or migrate in the second year, i.e. 985–14.775 = 970.225 contribute a full year of observation time in the second year. So the expected total number of person-years for the initial 1000 individuals for the second year is (970.225 + 14.775 × 0.5) = 977.612 person-years, i.e. 0.977612 per person. The general equation for the second year is thus

2 $$ {\hat{py}}_{i2 n}=\kern0.7em \left[\left(1-{\gamma}_{i2}\right)+0.5{\gamma}_{i2}\right] P\left(\mathrm{survive}\ \mathrm{year}\ 1\right)=\left(1-0.5{\gamma}_{i2}\right)\left(1-{\gamma}_{i1}\right) $$

This procedure continues for an arbitrary year k until the end-of-follow-up-date as

3 $$ {\hat{py}}_{i k n}=\kern0.7em \left(1-0.5{\gamma}_{i k}\right){\displaystyle \prod_{\kappa =1}^{k-1}\left(1-{\gamma}_{i\kappa}\right)} $$

This can be rewritten as

4 $$ {\hat{py}}_{i k n}=\kern0.7em \left(1-0.5{\gamma}_{i k}\right)\left(1-{\gamma}_{i, k-1}\right){\displaystyle \prod_{\kappa =1}^{k-2}\left(1-{\gamma}_{i\kappa}\right)}=\kern0.5em \frac{\left(1-0.5{\gamma}_{i k}\right)\left(1-{\gamma}_{i, k-1}\right)}{\left(1-0.5{\gamma}_{i, k-1}\right)}{\hat{py}}_{i, k-1, n} $$

Since $\frac{\left(1-0.5{\gamma}_{i k}\right)\left(1-{\gamma}_{i, k-1}\right)}{\left(1-0.5{\gamma}_{i, k-1}\right)}$ is numerically close to (1 − *γ*_*i*,*k*_) unless the rates change strongly from one year to the next, we use the simpler equation

5 $$ {\hat{py}}_{i kn}=\kern0.6em \left(1-{\gamma}_{i j}\right){\hat{py}}_{i, k-1, n} $$

A full numerical example is given in Table 1. In a cohort individual entry dates and birth dates are arbitrary,, and the calendar period changes over time and so do the corresponding rates μ_ij_ and ν_ij_.. We now use the common notation for person-years which are given according to age groups. In praxis, 5-year age groups i = 1, .., I are commonly used. We use this grouping in the following. The expected person-time that an individual n contributes to the age class i × calendar year j, j = 1, …, J , ${\hat{py}}_{ijn}$ is then given as the probability that the class is entered multiplied by the expected observation time within that class. The probability that the class is entered is the product of the failure probabilities in all previous age × calendar year classes (i’,j’), i.e.

**Table 1:** Numerical example: expected person-year contributions and calculation procedure for a single individual

		Rates (per 1000)			E(py_j_)			Sensitivity analysis E(py_j_)	
Year (j)	Age (i)	μ_ij_	ν_ij_	λ_ij_		Var(py_j_) (fixed rates)	Var(py_j_) (variable rates)	Factor for μ_ij_ 0.5	and ν_ij_ 1.5
1990	40	2	13	0.5	0.992	0.004	0.01725	0.9925	0.9775
1991	41	2	13	0.5	0.978	0.019	0.03384	0.9851	0.9558
1992	42	2	13	0.5	0.963	0.035	0.04979	0.9777	0.9449
1993	43	2	13	0.5	0.949	0.050	0.06513	0.9704	0.9238
1994	44	2	13	0.5	0.934	0.066	0.07988	0.9632	0.9031
1995	45	3	10	0.8	0.921	0.078	0.09252	0.9569	0.8840
1996	46	3	10	0.8	0.908	0.087	0.1047	0.9507	0.8670
1997	47	3	10	0.8	0.896	0.097	0.11655	0.9446	0.8500
1998	48	3	10	0.8	0.884	0.106	0.12798	0.9385	0.8335
1999	49	3	10	0.8	0.872	0.114	0.13903	0.9324	0.8173
2000	50	5	7	1.2	0.860	0.122	0.14909	0.9268	0.8008
2001	51	5	7	1.2	0.849	0.129	0.15884	0.9213	0.7854
2002	52	5	7	1.2	0.838	0.136	0.16830	0.9157	0.7700
2003	53	5	7	1.2	0.827	0.143	0.17748	0.9103	0.7552
2004	54	5	7	1.2	0.816	0.149	0.18638	0.9048	0.7404
Total	total				13.487	1.335	1.677	14.190	12.830

Date of birth:January 1^st^, 1950; Begin of follow-up: January 1^st^, 1990;

End of follow-up:December 31^st^, 2004

μ_ij_ - mortality rate (all causes other than disease of interest), age i, year j

ν_ij_ - migration rate, age i, year j

λ_ij_ - incidence rate (disease of interest), age i, year j

$$ {\displaystyle \prod_{\begin{array}{l}\kern1.5em  i\hbox{'}, j\hbox{'},\\ {}\kern1em  i\hbox{'}< i, j\hbox{'}< j\\ {}\left( i\hbox{'}, j\hbox{'}\right)\ne \left( i, j\right)\\ {}\end{array}}\left(1-{\gamma}_{i\hbox{'} j\hbox{'}} p{y}_{i\hbox{'} j\hbox{'} n}^{*}\right)} $$, where *γ*_*i* ' *j* '_is the sum of the mortality and migration rate in class (i,j) and *py*_*i* ' *j* ' n_^*^ is the maximal person-time (raw person-years) in that class (which is 1 year under this categorization), and thus we get

6 $$ {\hat{py}}_{i j n}=\left({\displaystyle \prod_{\begin{array}{l}\kern1.5em  i\hbox{'}, j\hbox{'},\\ {}\kern1em  i\hbox{'}< i, j\hbox{'}< j\\ {}\left( i\hbox{'}, j\hbox{'}\right)\ne \left( i, j\right)\end{array}}\left(1-{\gamma}_{i\hbox{'} j\hbox{'}} p{y}_{i\hbox{'} j\hbox{'} n}^{*}\right)}\right) p{y}_{i j n}^{*}\left(1-0.5{\gamma}_{i j}\right) $$

Equation (6) is the general form of Eq. (3) which allows arbitrary entry times, birth dates, and class length. Summing up the expected person-years for each cohort member over all age-groups and calendar years then gives an estimate for the expected person-years in all categories, ${\hat{py}}_{ij}={\displaystyle \sum_{n=1}^N{\hat{py}}_{ij n}}$. The person-years from the cases *py*_*ij*,*cases*_ have to be added. An estimate for the SIR is then given by

7 $$ SIR\kern0.5em =\kern0.5em \frac{O}{{\displaystyle \sum_{i=1}^I{\displaystyle \sum_{j=1}^J\left(\widehat{p{y}_{i j}}+ p{y}_{i j, cases}\right)\kern0.5em {\lambda}_{i j}}}} $$

### Computational aspects

In principle, the person-years contributions for each individual for each calendar year and age class can be calculated separately, and thus the estimate in Eq. (6) can be used directly. Computationally, it is simpler to first calculate the person-years by age group and calendar period under the assumption *μ*_*ij*_ = *ν*_*ij*_ = 0, i.e. for all individuals not diseased the study endpoint is used as end of follow-up date for all individuals. Common software to calculate person-years is applied here and gives the ‘raw’ person-years which we have denoted as *py*_ij_^*^. This also is the upper bound for the total person-years and can be used to calculate the minimal SIR. We apply the above procedure iteratively from the first up to the last year of follow-up as

8 $$ {\widehat{py}}_{i1}= p{y}_{i1}^{*}\kern0.24em \left(1-{\gamma}_{i1}\right) $$

similar as in Eq. (5). Here *i* is the index for age group and 1 the first calendar year to which individual(s) contribute person-times.

If the population did not undergo aging, the adjusted person-years *py* in the second calendar year would be

9 $$ {\widehat{py}}_{i2}=\left( p{y}_{i2}^{*}-\left( p{y}_{i1}^{*}-{\widehat{py}}_{i1}\right)\right)\kern0.24em \left(1-{\gamma}_{i2}\right) $$

since to the raw py in year *2* contribute individuals who enter the cohort in year *2* and individuals who entered in year *1*. For the latter group, the expected loss of py in the first year, $p{y}_{i1}^{*}-{\widehat{py}}_{i1}$, must be taken into account and subtracted from the raw py in year *2*. On this adjusted term, $\left( p{y}_{i2}^{*}-\left( p{y}_{i1}^{*}-{\widehat{py}}_{i1}\right)\right)$, the rates for the year *2* (1 − *γ*_*i*2_) must be applied. This procedure continues until the last calendar year is reached for which observation times are recorded.

In the iterative process we must take aging into account, i.e. people move through the underlying age groups. Since mortality rates are commonly provided in five year age groups we use this categorization as well. For a given year, the observation times of a five-year age group undergo aging such in expectation that one fifth will move to the next oldest five-year age group in the next year. The estimated person-years for the second calendar year *j2* of age group i, ${\widehat{py}}_{i2}$ is therefore the raw person-years, *py*_*i*2_^*^, minus 4/5 of the expected loss in the same age group, minus 1/5 of the expected loss in the next youngest age group, yielding

10 $$ \begin{array}{l}{\widehat{py}}_{i2}=\kern0.62em \Big[ p{y}_{i\kern0.1em 2}^{*}-\left( p{y}_{i\kern0.1em 1}^{*}-{\widehat{py}}_{i\kern0.1em 1}\right)\kern0.22em \frac{4}{5}\kern0.2em \\ {}\kern3.1em -\left( p{y}_{i- 1,1}^{*}-{\widehat{py}}_{i- 1,1}\right)\frac{1}{5}\Big]\kern0.34em \left(1-{\gamma}_{i2}\right)\;\end{array} $$

Finally, for an arbitrary year j we get

11 $$ \begin{array}{l}{\widehat{py}}_{i, j}=\kern0.62em \Big[ p{y}_{i, j}^{*}-\left( p{y}_{i, j-1}^{*}-{\widehat{py}}_{i, j-1}\right)\kern0.22em \frac{4}{5}\kern0.2em \\ {}\kern3.1em -\left( p{y}_{i- 1, j-1}^{*}-{\widehat{py}}_{i- 1, j-1}\right)\frac{1}{5}\Big]\kern0.34em \left(1-{\gamma}_{i j}\right)\;\end{array} $$

We have written an SAS Macro to perform the required calculations (available as Additional file 1). As mentioned before, the exact solution requires the procedure for the non-diseased only and adding the (known) person-years from the diseased (the cases).

### Variance estimation and confidence intervals

Standard procedures for SIR confidence intervals consider the observed number of cases O as random variable which is Poisson distributed and the denominator as fixed. Our procedure to estimate the accumulated person-years adds a variance component to the expected number of deaths E, and therefore an additional variance component must be taken into account. In the following we show that this component is so small compared to the component in the numerator that it can be neglected.

The delta-method asymptotically gives $V a r\left(\frac{O}{E}\right)=\frac{1}{E^2} V a r\kern0.5em  O+\frac{O^2}{E^4} V a r\kern0.5em  E$. Since *O* is Poisson distributed, its variance is equal to the expectation, i.e. Var(*O) = E(O).* For *Var (E)* it holds:

12 $$ V a r(E)= V a r\left({\displaystyle \sum_{i=1}^I{\displaystyle \sum_{j=1}^J\widehat{p{ y}_{i j}}\kern0.5em {\lambda}_{i j}}}\right)=\left({\displaystyle \sum_{i=1}^I{\displaystyle \sum_{j=1}^J{\left({\lambda}_{i j}\right)}^2 Var\Big(\widehat{p{ y}_{i j}\Big)}}}\right) $$

The evaluation of $V a r\Big(\widehat{p{ y}_{ij}\Big)}$ is done under the same assumptions on entry times and ages as before. For the first year (1) and for a single individual n, the variance of its person-year contribution is $V a r{\overset{\wedge }{\Big( py}}_{i1 n}\Big)=\left(\frac{1}{3}-\frac{\gamma_{i1}}{4}\right){\gamma}_{i1}$. This is shown as follows: Let *Y* denote the random variable denoting the (unknown) person-time for an individual in the first year of observation. Its density function is $$ {f}_Y(y)=\left\{\begin{array}{c}\hfill 1-{\gamma}_{i1}\hfill \\ {}\hfill {\gamma}_{i1}\hfill \end{array}\right.\kern1em  if\kern1em \begin{array}{c}\hfill Y=1\hfill \\ {}\hfill Y\in \left(0,1\right)\hfill \end{array} $$ , assuming a uniform distribution given an event (migration or death from other cause) occurred. Then we get expectation *E*(*Y*) = 1 × (1 − *γ*_*i*1_) + 0.5 × *γ*_*i*1_ = 1 − 0.5*γ*_*i*1_. We are interested in the variance of Y. We use the relation *Var*(*Y*) = *E*(*Y* ^2^) − [*E*(*Y*)]^2^. For *f*_*Z*_(*y*), *Z* := *Y* ^2^ we get $$ {f}_Z(z)=\left\{\begin{array}{c}\hfill 1-{\gamma}_{i1}\hfill \\ {}\hfill 1/\left(2\sqrt{z}\right)\hfill \end{array}\right.\kern1em  if\kern1em \begin{array}{c}\hfill Z=1\hfill \\ {}\hfill Z\in \left(0,1\right)\hfill \end{array} $$ and then after basic probability theory *E*(*Z*) = (1 − *γ*_*i*1_) + *γ*_*i*1_/3 and thus *Var*(*Y*) = (1 − *γ*_*i*1_) + *γ*_*i*1_/3 − (1 − *γ*_*i*1_/2)^2^ = *γ*_*i*1_/3 − *γ*_*i*1_^2^/4 = *γ*_*i*1_(1/3 − *γ*_*i*1_/4).

For small rates, this is smaller than the binomial variance *γ*_*i*1_(1 − *γ*_*i*1_)by a factor of about 3. This follows intuitively since individuals with an event contribute between zero and one person-years in the year of the event, not zero. Then, for all individuals in this year and age group i, we have $V a r{\overset{\wedge }{\Big( py}}_{i1}\left)={\overset{\wedge }{\Big( py}}_{i1}\right)\left(\frac{1}{3}-\frac{\gamma_{i1}}{4}\right){\gamma}_{i1}$ . For the second year, we have for a single individual $V a r{\overset{\wedge }{\Big( py}}_{i2 n}\Big)=\left(1-{\gamma}_{i2}\right){\gamma}_{i2}+\left(\frac{1}{3}-\frac{\gamma_{i2}}{4}\right){\gamma}_{i2}$ . In order to show that the variance component from the estimated person-years is negligible it is sufficient to consider an upper bound of the total variance of the expected number of cases, E. and for all individuals in the second year and age group i, we have as an upper bound $V a r{\overset{\wedge }{\Big( py}}_{i2}\left)={\overset{\wedge }{\Big( py}}_{i2}\right)\left(1+{0.5}^2\right)\left(1-{\gamma}_{i2}\right){\gamma}_{i2}$

Summing up, we have as an upper bound

$$ V a r(E)=\left({\displaystyle \sum_{i=1}^I{\displaystyle \sum_{j=1}^J{\left({\lambda}_{i j}\right)}^2 Var\Big(\widehat{p{ y}_{i j}\Big)}}}\right)=\left({\displaystyle \sum_{i=1}^I{\displaystyle \sum_{j=1}^J{\left({\lambda}_{i j}\right)}^2\widehat{p{ y}_{i j}}\left( j-1+{0.5}^2\right)\left(1-{\gamma}_{i j}\right){\gamma}_{i j}}}\right) $$

since $\overset{\wedge }{\lambda_{ij} p{y}_{ij}}={E}_{ij}$,the expected number of cases in age group × calendar period (*i*, *j*), we write

13 $$ V a r(E)=\left({\displaystyle \sum_{i=1}^I{\displaystyle \sum_{j=1}^J{E}_{i j}{\lambda}_{i j}\widehat{p{ y}_{i j}}\left( j-1+{0.5}^2\right)\left(1-{\gamma}_{i j}\right){\gamma}_{i j}}}\right) $$

Therefore under the rare disease assumption *λ*_*ij*_ and *γ*_*ij*_small, the variance of the denominator is several magnitudes smaller than the variance of the numerator. Additional aspects on variance estimation can be found in Additional file 2. The appropriateness of this method has additionally been checked by simulation using the numerical example below. The evaluation shows that the value resulting from Eq. (13) is very low, and that the variance is very small compared to the variance of the numerator. Therefore, it can be neglected and standard methods to calculate the confidence interval can be used.

### Sensitivity analysis

The method developed provides an estimate for the person-years and hence for the SIR if the mortality rates and the migration rates are applicable to the cohort. Since the question may arise whether the mortality rates from the underlying population are applicable to the cohort and whether the migration rates are reliably estimated, the question of bias may be more relevant than the additional variance induced by the person-years estimation. If *γ*_*ij*_ is underestimated, the person-years are overestimated, up to the maximum when *γ*_*ij*_ is zero. Conversely, if *γ*_*ij*_ is overestimated, the person-years are underestimated. We recommend to perform a detailed sensitivity analysis by estimating the SIR with reasonably modified migration and mortality rates (see example below). As will be seen in the study example (section 4), the SIR estimate is relatively robust against violations of the assumptions. If migration rates are reliably estimated, however subject to random variation, an additional variance component can easily be incorporated (see numerical example below).

## Results

### A numerical example

A numerical example is given in Table 1 to show the effect of the estimation procedure on the person-years allocation in the (calendar year) × (age) cross-classification. We consider one single individual *n* which entered the cohort on January 1st, 1990. This person did not become an incident case during the follow-up period. Therefore, death from another cause or out-migration out of the catchment area of the registry could have occurred at any date during the follow-up period with death rates *μ*_*ij*_ and migration rates *ν*_*ij*_, depending on calendar year and age. We assume different migration and mortality rates in different age groups. According to this example, this person with an unknown follow-up status contributes an expected value of *py*_*n*1_ =0.992 person-years to the first year of follow-up, *py*_*n*2_ =0.978 person-years to the second, and *py*_*n*15_ =0.816 person-years to the last year of follow-up instead of a maximal value of one person-years per year. The expected total number of person-years for this person is 13.487 instead of a maximal possible value of 15 years. These are realistic rates and yield a reduction of person-years of 10.1%. If *γ*_*ij*_ are assumed fixed, the Variance of *py*_(*n*)(1)_ is approximately 0.5^2^ *γ*_*ij*_(1 − *γ*_*ij*_)= 0.004, the variance of *py*_*n*2_ and *py*_*n*3_ is approximately (1 + 0.5^2^)*γ*_*ij*_=0.019, and (2 + 0.5^2^)*γ*_*ij*_=0.035, and so on. The variance of the total person-years estimate for this individual is 1.335. If *γ*_*ij*_ are random with variance var(*γ*_*ij*_) = *σ*_*γ*_^2^, we get var(*py*_*n*1_)= *γ*_*ij*_(1 − *γ*_*ij*_) + *σ*_*γ*_^2^. The variance component from the rates is here assumed as *σ*_*γ*_^2^ = 0.05^2^. Then the variance of the total person-years estimate for this individual is 1.667, an increase of 22%, however, still so small that it can be neglected.

It would be incorrect to assign to this individual 13.429 person-years from the begin of follow-up since this would wrongly yield to a fixed loss-to-follow-up date in the year 2003, and hence full years of observation for all previous years, and zero person-years for the year 2004.

In Additional file 3 we expand this numerical example by assuming that we have a cohort of 1000 individuals with the same characteristics as the individual above. It shows numerically that the variance component induced by the person-years estimate in the denominator of the SIR is small compared to the variance component in the numerator so that it can be neglected. The sensitivity analysis for this dataset used rates with factors 1.5 and 0.75 yielded a bias in the SIR of only 7%. It shows that the procedure is relatively robust against violations in the rate estimates.

### Simulation study

A simulation study has been performed to investigate the performance of the estimation procedure. The setup of the simulation is as follows: A cohort of size *N =* 1000 was simulated with parameters as in the preceding numerical example. For each dataset, the number of observable events (incident cases) and unobservable losses (deaths from other causes or out-migration) and the corresponding failure time of the events and time of loss was simulated. The exact number of person-years was recorded and compared with the estimated number of person-years according to the above procedure. The main parameter for comparison was the relative difference of observed and exact person-years, i.e. ${\varDelta}_{p y\_ rel}=\frac{p{y}_{exact}- p{y}_{estimated}}{p{y}_{exact}}$.

According to the chosen parameters we observed between 3 and 23 events (median 11, mean 10.75) and between 145 and 218 losses from deaths or out-migration within the 15 year observation period (median 179, mean 179.7). The simulation results regarding the observed and estimated py as well as the absolute and relative difference and number of events are given in Table 2. We observe a slight underestimation of the true person-years of 0.4% with an empirical 90% confidence interval as (−1.0% – 1.9%). We conclude that the procedure is sufficiently accurate. In comparison we would find a mean overestimation of the person-years of 10.7% if all individuals are wrongly assumed to remain under risk.

**Table 2:** Simulation study^a^. Comparison of exact and estimated person-years

Parameter	Mean	Standard deviation	Median	Interquartile range
Person-years (exact)	13466.0	114.7	13467.1	13390.0 – 13544.2
Person-years (estimated)	13409.2	44.3	13408.5	13381.4 – 13435.6
Person-years (biased, if deaths/migration ignored)	14905.3	31.9	14907.2	14884.9 – 14926.9
Number of events	10.95	3.26	11	9 – 13
Number of deaths or migrants	179.7	179	179	172 - 188
Difference exact – estimated person - years	56.8	116.9	58.5	−21.15 – 134.2
Relative difference exact – estimated (in %)	0.41	0.86	0.43	−0.16 – 0.99

^a^Parameter for the simulation

Number of simulation runs 1000

Number of observations 1000

Follow-up duration 15 years

Incidence rates (variable, see table 1)

Mortality rates (variable, see table 1)

Migration rates (variable, see table 1)

The simulation program was written in SAS and is listed in the supplemental material.

### Application

A cohort study was performed with 18621 migrants from the Former Soviet Union who immigrated between 1990 and 2005 to the German federal state of Saarland. For every individual name, sex, date of birth, date of immigration (start of the observation period), and first city of residence was available. A complete incidence follow-up was done by linking the cohort with the Saarland cancer registry which is a high quality register for this federal state [5]. 470 incident cancer cases (except non-melanoma skin cancer) were identified. Mortality follow-up and tracing of individuals who relocated from the state was not performed.

Age, sex and calendar-year specific “raw” person-years were calculated at the end of the observation period (31^st^ December 2005) by calendar year and 5-year age groups. For individuals with cancer diagnosis, the date of diagnoses was set as the endpoint of their observation time. A summarized result is given in Table 3.

**Table 3:** Raw and adjusted person-years, Cohort study in Saarland, Germany, 1990-2005

	Raw person-years				Adjusted person-years			
	Calendar period				Calendar period			
Age	1990–1994	1995–1999	2000–2005	Total	1990–1994	1995–1999	2000–2005	Total
0 – 4	2435.1	5111.7	3459.0	11005.8	2247.5	4753.9	3115.3	10116.6
5 – 9	1618.1	5844.4	7102.9	14565.4	1467.5	5363.9	6564.7	13396.1
10 – 14	1670.8	5352.9	9988.2	17011.8	1516.5	4873.0	9285.1	15674.6
15 – 19	1521.3	4928.9	9379.4	15829.7	1372.4	4470.7	8628.2	14471.3
20 – 24	1876.9	4221.0	8320.0	14417.9	1686.7	3760.0	7580.1	13026.7
25 – 29	2724.3	5295.8	6974.0	14994.1	2464.6	4704.2	6207.7	13376.4
30 – 34	2802.5	7195.7	8072.7	18071.0	2583.7	6608.9	7221.2	16413.8
35 – 39	1864.9	6733.4	10915.2	19513.5	1718.6	6238.8	10011.3	17968.8
40 – 44	635.5	4408.0	10522.7	15566.2	573.2	4085.4	9707.7	14366.2
45 – 49	973.1	1631.1	7272.1	9876.2	890.3	1429.1	6667.3	8986.7
50 – 54	1145.8	2608.9	2883.5	6638.2	1071.9	2328.1	2408.7	5808.7
55 – 59	1035.9	2806.0	3706.3	7548.3	969.7	2501.0	2863.3	6647.3
60 – 64	926.1	2608.6	4174.2	7708.8	865.2	2291.1	3575.4	6731.7
65 – 69	422.2	1907.7	3541.6	5871.6	386.9	1655.4	2922.3	4964.5
70 – 74	281	855.5	2713.2	3849.6	252.2	690.2	2169.6	3112.0
75 – 79	200.5	572.8	1154.5	1927.9	175.3	432.3	758.8	1366.4
80 – 84	93.4	394.8	761.9	1250.2	76.0	273.2	421.1	770.4
85+	19.3	199.5	845.8	1064.6	13.2	109.4	430.2	552.8
Total	22246.7	62676.7	101787.2	186710.8	20331.3	56568.5	90851.3	167751.1

Adjustment of the person-years was done according to Eqs. (1) and (2). The German mortality rates for all causes of death except cancer within the cohort (μ_ij_) were used. These were obtained from WHO mortality database [6]. The migration rates (ν_ij_) were taken from a partial follow-up and from a similar study on migrants in another federal state [7]. This is considered the most appropriate model. The rates are given in Table 4 (model 1). The cancer incidence rates λ_ij_ were taken from the Saarland cancer registry.

**Table 4:** Yearly migration rates for three different models, Cohort study in Saarland, Germany, 1990–2005

	Migration rates ν_ij_								
	Before 1993			1993 to 1996			After 1996		
	Age <30	Age 30–50	Age >50	Age <30	Age 30–50	Age >50	Age <30	Age 30–50	Age >50
Model 1 (external estimate)	6.5%	5.5%	2.5%	2.2%	1.1%	2.5%	0.4%	0.4%	0.8%
Model 2 (two times lower than model 1)	3.25%	2.75%	1.25%	1.1%	0.55%	1.25%	0.2%	0.2%	0.4%
Model 3 (two times higher than model 1)	13.0%	11.0%	5.0%	4.4%	2.2%	5.0%	0.8%	0.8%	1.6%

Table 5 gives the “raw” and adjusted person-years for the 5-year age groups and calendar periods for the cohort. The overall cancer SIR is 1.11 in males and 1.01 in females. Using the “raw” person-years for calculating E, the SIR would be 0.90 and 0.86. The difference between “raw” and estimated total person-years was 18959.7 person-years, or 11.3%.

**Table 5:** Estimated person-years and expected cancer cases, and SIR according to different models, Cohort study in Saarland, Germany, 1990–2005

		Male					Female			
	Observed	Expected SIR 95% confidence interval				Observed	Expected SIR 95% confidence interval			
		Model 1	Model 2	Model 3	Model 4		Model 1	Model 2	Model 3	Model 4
Person-years		81,193.4	74,377.9	84,907.6	90,510.7		86,557.7	79,308.5	90,505.8	96,200.2
All Cancers^a^	235	211.0 1.11 0.98, 1.27	189.0 1.24 1.09, 1.41	226.2 1.04 0.91, 1.18	261.1 0.90 0.79, 1.02	235	232.0 1.01 0.89, 1.15	209.7 1.12 0.99, 1.27	244.2 0.96 0.85, 1.09	274.5 0.86 0.75, 0.97
Stomach	27	8.3 3.25 2.14, 4.75	7.5 3.60 2.37, 5.26	8.8 3.07 2.02, 4.48	10.5 2.57 1.68, 3.75	21	6.8 3.09 1.91, 4.73	6.1 3.44 2.13, 5.27	7.1 2.96 1.83, 4.53	8.4 2.50 1.55, 3.83
Colorectal	18	31.0 0.58 0.34, 0.92	27.6 0.65 0.39, 1,03	32.8 0.55 0.33, 0.87	39.1 0.46 0.27, 0.73	39	31.9 1.22 0.87, 1.67	28.6 1.36 0.97, 1.86	33.6 1.16 0.83, 1.59	39.2 0.99 0.71, 1.36
Lung	61	36.2 1.69 1.31, 2.17	32.4 1.88 1.46, 2.42	38.3 1.59 1.24, 2.05	45.0 1.36 1.05, 1.74	6	15.7 0.38 0.14, 0.83	14.1 0.43 0.16, 0.93	16.6 0.36 013, 0,79	18.5 0.32 0.12, 0.71
Breast						57	72.1 0.79 0.61, 1.02	65.3 0.87 0.67, 1.13	75.8 0.75 0.58, 0.97	83.6 0.68 0.53, 0.88
Prostate	35	37.6 0.93 0.65, 1.29	33.3 1.05 0.73, 1.46	40.0 0.88 0.61, 1.22	48.7 0.72 0.50, 1.00					
Leukaemia	9	5.9 1.53 0.70, 2.90	5.4 1.67 0.76, 3.17	6.3 1.43 0.65, 2.71	7.1 1.27 0.58, 2.41	7	5.2 1.35 0.54, 2.77	4.7 1.49 0.60, 3.07	5.5 1.27 0.51, 2.62	6.2 1.13 0.45, 2.33

^a^except nonmelanoma skin cancer

Model 1– migration based on available data

Model 2 – assuming doubled migration of model 1

Model 3 – assuming halved migration of model 1

Model 4 – raw person-years

The sensitivity analysis is done by estimating the number of person-years with different assumptions on migration rates. Model 2 uses halved and model 3 doubled rates (see Table 4). Results of the estimation procedure in terms of person-years and expected number of cancer cases are shown in Table 5 for selected cancer sites based on Saarland incidence rates [8]. Relatively little variation between the different models is observed. The biggest difference to model 1 for the expected number of cases is seen for model 2 in all female cancers with 22.3 cases. For other cancer sites the maximum differences (found between models 1 and 2) in incident cancer cases are 4.3 and 6.8, respectively. Sensitivity analysis demonstrates that estimated expected number of cancer cases and therefore corresponding SIR are quite robust with regard to the underlying assumptions. SIR for different cancer sites according to different models are also shown in Table 5 with the respective standard 95% confidence intervals. Model 4 gives the lower limit of the SIR estimates using the raw person-years for calculating the expected number of cases. On average, the expected number of cases are about 25% higher in this model.

## Discussion

We have presented a method to estimate the standardized incidence ratio in a cohort study for which a linkage with a disease registry has been performed, however, for which a mortality follow-up including tracing of individuals which moved out was not directly possible. This situation is not a rare one: cancer registries exist in many parts of the world, and disease registries for other diseases, such as diabetes, stroke, and others have been developed, e﻿.﻿g. [9, 10]. A straightforward mortality follow-up is possible only in countries with a death register or comparable data resources, such as for example the US, Great Britain and the Scandinavian countries. For many other countries, such as Germany, Switzerland, France, Italy, Spain and others the method presented here is an alternative to the sometimes heavily bureaucratic mortality follow-up procedures. Epidemiologists use these registries to assess disease incidence, and a linkage of cohorts with these registries is technically straightforward. We showed that under quite robust assumptions it is possible to estimate the accumulated number of person-years, and provided a method to calculate the SIR and its 95% confidence intervals taking into account the estimation procedure.

In our example, we present the analysis of cancer incidence in a cohort by making assumptions on mortality from other causes and on migration. First, the same mortality as the German population within the cohort was taken. Results from a similar cohort show that this assumption may be reasonable [4]. A slight underestimate of the number of person-years is possible, though. The second assumption refers to migration. There are good estimates on migration by age group from other cohort studies in Germany, and also on migrants from the former Soviet Union who migrated to another state in Germany. We think these rates can be applied here, however, some uncertainty will remain. A small subset of the cohort (about 20%) has been followed-up by now, and results support our assumption.

Here, we have considered cancer as the disease of interest which can be regarded as a rare disease in terms of the yearly incidence rates. If the disease is common, then the time from begin of observation until disease onset is shorter, and then for more individuals the raw person-time is the correct person-time. Consequently, the bias when using the raw person-years becomes smaller.

Our sensitivity analyses were reassuring in that respect. SIR estimates showed relatively little variation with modified rate assumptions. Still, a bias in the estimated expected value of cases is possible, and this appears to play a bigger role than the increase of variance by the estimation procedure. We showed that the additional variance induced by variation in the denominator is small relative to the variation in the number of observed cases. This allows using standard method of inference, in particular the application of exact confidence intervals if the observed number of cases is small.

The completeness of the registry is another relevant issue. In the case of the cancer registry in the Saarland, the percentage of DCO (death certificate only) cases in the registry is very low, and all those cases within the cohort are likely to have been identified with the linkage procedure.

## Conclusions

We showed that valid estimation of the observation time is possible for cohort studies with incomplete follow-up data. This is especially relevant for cohort studies where a complete assessment of the individual vital status is not easily possible but which can be linked to disease registries in order to assess the number of incident cases within the cohort.

## Additional files

Additional file 1: py estimation macro for SAS. (DOCX 17 kb)

Additional file 2: Addition aspects on variance estimation. (DOCX 21 kb)

Additional file 3: Numerical example showing py and variance estimation. (DOCX 37 kb)

## Abbreviations

py: person-years
RR: Rate ratio
SIR: Standardized incidence ratio
SMR: Standardized mortality ratio
