# Supplementary material for: Estimating the standardized incidence ratio (SIR) with incomplete follow-up data
Source: BMC Med Res Methodol. 2017 Apr 12;17:55. doi: 10.1186/s12874-017-0335-3 (PMC5389158; doi:10.1186/s12874-017-0335-3)
Supplement: Supplementary file 1 — py estimation macro for SAS. (DOCX 17 kb) [file 12874_2017_335_MOESM1_ESM.docx]

**data** example; input Agegroup CalendarYear Migration sex Rate PYraw;

cards ;

1 1990 0.065 2 0.001508184 13.557

2 1990 0.065 2 0.000168431 6.634

3 1990 0.065 2 0.000142721 11.327

4 1990 0.065 2 0.000289275 20.432

5 1990 0.065 2 0.000366065 23.048

6 1990 0.065 2 0.00035775 28.367

7 1990 0.055 2 0.000456241 21.146

8 1990 0.055 2 0.00065289 10.598

9 1990 0.055 2 0.000856856 6.643

10 1990 0.055 2 0.001301836 14.149

11 1990 0.025 2 0.001809716 10.286

12 1990 0.025 2 0.003064593 12.995

13 1990 0.025 2 0.005385376 9.173

14 1990 0.025 2 0.009818167 5.601

15 1990 0.025 2 0.018192305 4.623

16 1990 0.025 2 0.038151164 3.082

17 1990 0.025 2 0.073659665 0.013

18 1990 0.025 2 0.163726364 0

1 1991 0.065 2 0.001359517 103.057

2 1991 0.065 2 0.000136993 75.597

3 1991 0.065 2 0.000119617 86.643

4 1991 0.065 2 0.000302506 93.823

5 1991 0.065 2 0.000317823 133.039

6 1991 0.065 2 0.00034578 172.092

7 1991 0.055 2 0.000477163 148.153

8 1991 0.055 2 0.000635567 83.905

9 1991 0.055 2 0.000875059 31.424

10 1991 0.055 2 0.001256442 78.271

11 1991 0.025 2 0.001805085 62.773

12 1991 0.025 2 0.002915629 64.489

13 1991 0.025 2 0.005198605 56.838

14 1991 0.025 2 0.009423052 32.298

15 1991 0.025 2 0.017282036 27.427

16 1991 0.025 2 0.036478312 15.867

17 1991 0.025 2 0.070997307 4.916

18 1991 0.025 2 0.158432689 0.254

1 1990 0.065 1 0.00197895 20.056

2 1990 0.065 1 0.000207372 6.721

3 1990 0.065 1 0.000214174 15.466

4 1990 0.065 1 0.000766011 19.946

5 1990 0.065 1 0.001023627 28.376

6 1990 0.065 1 0.001036966 30.877

7 1990 0.055 1 0.001320288 25.355

8 1990 0.055 1 0.00174274 12.862

9 1990 0.055 1 0.002215547 7.836

10 1990 0.055 1 0.003456001 12.325

11 1990 0.025 1 0.005166574 11.474

12 1990 0.025 1 0.008379435 9.35

13 1990 0.025 1 0.01297867 2.178

14 1990 0.025 1 0.02108158 2.757

15 1990 0.025 1 0.033807317 1.379

16 1990 0.025 1 0.060690554 0

17 1990 0.025 1 0.103788291 0.18

18 1990 0.025 1 0.188092221 0

1 1991 0.065 1 0.001740527 138.064

2 1991 0.065 1 0.00018245 72.552

3 1991 0.065 1 0.000172238 98.047

4 1991 0.065 1 0.000771507 85.805

5 1991 0.065 1 0.001061707 128.681

6 1991 0.065 1 0.001107148 154.873

7 1991 0.055 1 0.001360351 161.968

8 1991 0.055 1 0.001832302 93.689

9 1991 0.055 1 0.002365925 36.645

10 1991 0.055 1 0.003442059 66.134

11 1991 0.025 1 0.005119849 66.424

12 1991 0.025 1 0.007989056 52.203

13 1991 0.025 1 0.012908746 24.521

14 1991 0.025 1 0.019913385 17.864

15 1991 0.025 1 0.032260499 12.831

16 1991 0.025 1 0.059032377 2.047

17 1991 0.025 1 0.100186865 1.368

18 1991 0.025 1 0.18437138 0

;

**run**;

/*--------------------------------------------------------------------------------

| Adjusting person-years in cohorts with incomplete follow up data.

| V. Winkler & H. Ramroth

|

| Input dataset containing raw person-years and assumptions on migration

| and mortality by sex, calendarYear, and agegroup during follow up period.

|

| Short description of the needed macro variables needed:

|

| DataIn - input dataset: any formats will be deleted

| StartYr - first year for adjustment

| EndYr - last year for adjustment

| CalendarYear - calendaryear variable

| Sex - sex variable: has to be coded as 1 or 2

| Agegroup - agegroup variable: should be coded numerically starting with 1

| PYraw - variable contains raw person-years assuming neither mortality nor migrtion

| Mortality - variable contains assumed mortality

| Migration - variable contains assumed migration

| PYadj - output variable containing adjusted person-years

| (stored as an additional varible in the input dataset)

| DataOut: - specifies the final dataset which contains the original variables and the variable PYadj.

| If not specified, the input dataset will be taken.

|

| Comments to:

| Dr. Volker Winkler

| University of Heidelberg

| Institute of Public Health

| Im Neuenheimer Feld 324

| 69120 Heidelberg

| Germany

| tel +49 6221 56-38636

| volker.winkler@urz.uni-heidelberg.de

|

+-------------------------------------------------------------------------------*/

**%macro** StartPyCalc (DataIn, DataOut, StartYr, EndYr, CalendarYear, Sex, Agegroup, PYadj, PYraw, Mortality, Migration);

/*****************************************************************************************/

/*****************************************************************************************/

/*****************************************************************************************/

%IF "&DataIn"="" %THEN %LET DataIn = &syslast;

%IF "&StartYr"="" %THEN %DO;

%PUT >>>> ERROR: No starting year given ! <<<<;

%GOTO exit;

%END;

%IF "&DataOut"="" %THEN %LET DataOut = &DataIn;

/****************************************************************************************/

%PUT ------------------------------------------------------------;

%PUT ------------------------------------------------------------;

%PUT ------------------------------------------------------------;

%PUT ---;

%IF "&DataIn"="" %THEN %PUT ---> Input dataset: &syslast;

%ELSE %PUT ---> Input dataset: &DataIn.;

%PUT ---> PY adjustment will run: ;

%IF "&StartYr"="" %THEN %PUT ---> starting from NOT SPECIFIED;

%ELSE %PUT ---> starting from &StartYr;

%IF "&EndYr"="" %THEN %PUT ---> NOT SPECIFIED;

%ELSE %PUT ---> ending &EndYr;

%PUT ---> Results will be stored in Variable &PYadj. dataset: &DataOut.;

%PUT ---;

%PUT ------------------------------------------------------------;

%PUT ------------------------------------------------------------;

%PUT ------------------------------------------------------------;

/*****************************************************************************************/

/*****************************************************************************************/

/*****************************************************************************************/

proc sql NOPRINT;

select MIN(&AgeGroup.), MAX(&AgeGroup.) into :MinAgeGr , :MaxAgeGr

from &DataIn.

;

quit;

%LET TwiceAgeGr = %EVAL ( (&MaxAgeGr. - &MinAgeGr. + 1) * 2 );

data TmpDummy;

&sex.=**1**;

do &CalendarYear. = &StartYr. to &EndYr.;

do &AgeGroup. = &MinAgeGr. to &MaxAgeGr.;

output;

end;

end;

data TmpDummyf; set TmpDummy; &sex.=**2**;

data TmpDummy; set TmpDummy TmpDummyf;run;

proc sort data=TmpDummy;

by &CalendarYear. &sex. &Agegroup.;

proc sort data=&DataIn.;

by &CalendarYear. &sex. &Agegroup.;

run;

data &DataOut.;

format _all_;

retain &CalendarYear. &sex. &Agegroup.;

merge TmpDummy &DataIn. (where=(&StartYr. <= &CalendarYear. <= &EndYr.));

by CalendarYear sex Agegroup;

run;

data &DataOut.; set &DataOut.;

if &PYraw. = **.** then &PYraw.=**0**;

if &CalendarYear. = &StartYr. then &PYadj.=&PYraw. * (**1**-&Mortality.)*(**1**-&Migration.);

%LET StartYr=%EVAL(&StartYr. + 1);

%DO i = &StartYr. %TO &EndYr.;

PYr1=**.**;

PYr2=**.**;

PYr1=lag&TwiceAgeGr.( &PYraw.- &PYadj.);

if &sex.=**2** then PYr2=lag(PYr1);

if &sex.=**1** then PYr2=lag(PYr1);

if PYr2=**.** then PYr2=**0**;

if &CalendarYear.=&StartYr. then &PYadj.=( &PYraw.-PYr1***4**/**5**-PYr2***1**/**5**)*(**1**- &Mortality.)*(**1**- &Migration.);

if &PYadj.<**0** then &PYadj.=**0**;

%LET StartYr=%EVAL(&StartYr. + 1);

%END;

drop PYr1 PYr2;

run;

proc sql;

drop table TmpDummy, TmpDummyf;

quit;

%exit:

**%mend** StartPyCalc;

options nomprint nosymbolgen;

%***StartPyCalc*** ( DataIn = example ,

StartYr = **1990** ,

EndYr = **1991** ,

CalendarYear = CalendarYear,

Sex = Sex ,

Agegroup = Agegroup ,

PYadj = PYadj ,

PYraw = PYraw ,

Mortality = Rate ,

Migration = Migration

);
