# Supplementary material for: Estimating the standardized incidence ratio (SIR) with incomplete follow-up data
Source: BMC Med Res Methodol. 2017 Apr 12;17:55. doi: 10.1186/s12874-017-0335-3 (PMC5389158; doi:10.1186/s12874-017-0335-3)
Supplement: Supplementary file 2 — Addition aspects on variance estimation. (DOCX 21 kb) [file 12874_2017_335_MOESM2_ESM.docx]

**Variance estimation aspects**

Let *Y* denote the random variable denoting person-year for an individual in the first year of observation. Its density function is

$f_{Y}(y)=\left\{\begin{matrix} 1-p \\ p \end{matrix}\right. if \begin{matrix} Y=1 \\ Y\in(0,1) \end{matrix}$ , assuming a uniform distribution given an event occurred and denoting the probability of an event in the first year as p.

Then we get expectation $E\mathop{(Y}\limits)=1\times(1-p)+0.5\times p=1-0.5p$.

For the Variance of Y we use $Var\mathop{(Y}\limits)=E(Y^{2})-\left[E(Y)\right]^{2}$. For $f_{Z}(y), Z:=Y^{2}$ we get

$f_{Z}(z)=\left\{\begin{matrix} 1-p \\ 1/(2\sqrt{z}) \end{matrix}\right. if \begin{matrix} Z=1 \\ Z\in(0,1) \end{matrix}$ and then after basic probability theory $E(Z)=(1-p)+p/3$ and thus $Var(Y)=(1-p)+p/3-(1-p/2)^{2}=p/3-p^{2}/4.$

The simulation confirms this result. For the variance in the following years the calculation becomes more cumbersome, however, since an upper bound of the variance was shown to be so small that the variance component in the denominator can be neglected, we do not consider this further. The upper bound is obtained under the assumption that all individuals with an event contribute zero person-years for the year of the event.
