# Supplementary material for: Estimating the standardized incidence ratio (SIR) with incomplete follow-up data
Source: BMC Med Res Methodol. 2017 Apr 12;17:55. doi: 10.1186/s12874-017-0335-3 (PMC5389158; doi:10.1186/s12874-017-0335-3)
Supplement: Supplementary file 3 — Numerical example showing py and variance estimation. (DOCX 37 kb) [file 12874_2017_335_MOESM3_ESM.docx]

**Numerical example**

The numerical example of section 3 is further expanded here. We show numerically the result of the person-years estimation procedure and the subsequent effect of SIR estimation and SIR inference. We also show the result of the sensitivity analysis if the assumptions on migration resp. mortality rates are violated.

Assume we have a cohort of 1000 identical individuals as the single individual in Table 1 with a 15 year follow-up. Of these we assume K have been identified as incident cases through linkage with the disease registry at arbitrary dates within the follow-up period, and each of these k=1,.. K cases contribute person-years *py_observable_* with $py_{observable}:=\sum_{k=1}^{K} py_{k}$. Of the remaining 1000-K cohort members, the exact person-year contribution *py_unobservable_* is unknown. For these, as obtained from table 1, the expected person-years are (1000-K)×13.473. Adding the person-years of the observed incident cases (which are exactly known) we get as an estimate for the total number *py_total_ = py_unobservable_ +py_observable_ =(1000-K)×13.473 + py_observable_*  For K=0 we have an upper bound of the variances for the estimate of the total person-years and we get, given fixed resp. variable rates, values of 1335 and 1667, and the resulting approximate 95% confidence intervals are $13473\pm1.96\times\sqrt{\text{1000×}1335}$ $=(13401.4 , 13544.6)$and (13393.0,13553.0).

Assuming incidence rates λ for the disease of interest as given in Table 1 $\left(\lambda_{40-44,1990-1994}=0.5,\lambda_{45-49,1995-1999}=0.8,\lambda_{50-54,2000-2004}=1.2\right)$ per 1000 we get $Ê=(0.50\times4812+0.8\times4476+1.2\times4185)/1000=11.01$. This would yield an SIR of 15/11.01 = 1.35. Ignoring the variance of the denominator and using the crude normal approximation of O this yields a 95% confidence interval for the SIR of (15 +/- 1.96×√15)/11.01 = (15 +/- 1.96×3.873)/11.01 = 0.67-2.05.

The upper limit, assuming 15 years of follow- up for each individual, gives $Ê=12.5$. For the variance we get 174, 482, and 679 as

$Var(py_{40-44,1990-1994})=$174 = 1000×(0.004+0.019+0.035+0.050+0.066),

$Var(py_{45-49,1995-1999})=$482 and $Var(py_{50-54,2000-2004})=$ 679 and thus $Var(E)$=

$Var(py_{_{40-44,1990-1994}} \lambda_{_{40-44,1990-1994}}+py_{_{45-49,1995-1999}} \lambda_{_{45-49,1995-1999}}+py_{_{50-54,2000-2004}} \lambda_{_{50-54,2000-2004}})$= 174×(0.50/1000)^2^ + 482×(0.80/1000)^2^ + 679×(1.20/1000)^2^=0.00133. The 95% confidence interval for $Ê$ is $11.01\pm1.96\times\sqrt{.00133}=(10.93 , 11.07)$. The same calculation assuming the variance component for the rates yields a slightly larger variance of 0.0016. For the numerator, the variance depends on the observed number of cases. If, for example, we use O=15, we have $Var(O)$=15 from the Poisson distribution.

Thus, asymptotically $Var\left(\frac{O}{E}\right)=\frac{1}{E^{2}}Var O+\frac{O^{2}}{E^{4}}Var E=\frac{1}{11.01^{2}}15+\frac{15^{2}}{11.01^{4}}0.0013$=0.124+0.00002. Therefore, the variance component from the denominator is negligible in both cases under the given assumptions, and the standard procedure to calculate confidence intervals, ignoring the variance component in the denominator, can be applied. With the above numbers we get $S\hat{I}R=15/11.01=1.35$. The biased estimate, assuming 15 years of follow-up for all, yields $S\hat{I}R=15/12.5=1.20$, which is an underestimation. However, if reliable estimates in the rates are not available or the assumptions are questionable it is more relevant to carefully check the underlying assumptions regarding migration and mortality (see sensitivity analysis). If, for example, the rates are severely underestimated (by factor 0.75) or overestimated (by factor 1.5), the estimated expected number of person-years in periods 1990-1994, 1995-1999 and 2000-2004 are 4888, 4723, 4579 and 4675, 4215, 3840, respectively, yielding expected number of cases as $Ê=(0.50\times4888+0.8\times4723+1.2\times4579)/1000=11.72$ in case of rate underestimation and$Ê=(0.50\times4675+0.8\times4215+1.2\times3840)/1000=10.32$ in case of rate overestimation. with SIR estimates as 15/11.72=1.28 and 15/10.32=1.45. .
